# Induction of Protective Anti-CTL Epitope Responses against HER-2-Positive Breast Cancer Based on Multivalent T7 Phage Nanoparticles

**DOI:** 10.1371/journal.pone.0049539

**Published:** 2012-11-15

**Authors:** Somayeh Pouyanfard, Taravat Bamdad, Hamidreza Hashemi, Mojgan Bandehpour, Bahram Kazemi

**Affiliations:** 1 Department of Virology, Faculty of Medical Sciences, Tarbiat Modares University, Tehran, Iran; 2 Department of Medical Virology, School of Public Health, Tehran University of Medical Sciences, Tehran, Iran; 3 Biotechnology Department, School of Medicine, Shahid Beheshti University of Medical Sciences, Tehran, Iran; 4 Cellular and Molecular Research Center (CMRC), School of Medicine, Shahid Beheshti University of Medical Sciences, Tehran, Iran; Wayne State University School of Medicine, United States of America

## Abstract

We report here the development of multivalent T7 bacteriophage nanoparticles displaying an immunodominant H-2k^d^-restricted CTL epitope derived from the rat HER2/neu oncoprotein. The immunotherapeutic potential of the chimeric T7 nanoparticles as anti-cancer vaccine was investigated in BALB/c mice in an implantable breast tumor model. The results showed that T7 phage nanoparticles confer a high immunogenicity to the HER-2-derived minimal CTL epitope, as shown by inducing robust CTL responses. Furthermore, the chimeric nanoparticles protected mice against HER-2-positive tumor challenge in both prophylactic and therapeutic setting. In conclusion, these results suggest that CTL epitope-carrying T7 phage nanoparticles might be a promising approach for development of T cell epitope-based cancer vaccines.

## Introduction

Identification of tumor-associated antigens (TAAs) has facilitated rational design of anti-tumor vaccines. However, most currently defined TAAs are products of self-genes overexpressed by neoplastic tissues in the body [1]. This poses a significant challenge because effective cancer vaccine strategies should be able to by-pass tolerance to self-antigens. ErbB-2 (HER-2/neu) is a member of the epidermal growth factor receptor (EGFR) family that is often constitutively overexpressed and functions as an oncogene product in a substantial fraction of human breast cancers correlating with more aggressive tumor growth, greater invasiveness, enhanced metastatic potential and increased resistance to therapy [2]. The immunological tolerance to HER-2/neu has been demonstrated in previous studies. It has been shown that tolerance to self-antigens can be overcome by certain parts of the protein that can selectively activate the immune system without activation of suppressor T-helper cells [3]. In addition, regulatory authorities and also public opinion ask for ever safer and better characterized vaccines [4]. So, the use of the immunodominant epitopes instead of full-length proteins represents a potentially safer alternative to full-length proteins. This is particularly advantageous when targeting self-antigens such as HER-2 that mediate key biological functions in the body, as immune responses elicited by whole protein vaccines can stimulate the growth of tumor cells if the antibodies mimic the activity of growth factor ligands. Indeed, antibodies capable of stimulating the growth of HER-2-positive tumor cells have been reported [5], [6].

The identification of MHC class I (MHC-I)-binding peptides derived from TAAs has facilitated the development of T-cell epitope-based vaccines for cancer as reviewed by Van Der Bruggen *et al*. [7]. Although these vaccines possess a better safety profile; many obstacles remain in the rational design of peptide-based vaccines despite an increasing knowledge of the molecular recognition and stimulation of the immune system. Peptides administered alone are poorly immunogenic; therefore, enhancement of immunogenicity of the peptide vaccines through the use of adjuvants and delivery systems has been an active area of research for the development of peptide vaccines in recent years [8], [9].

Particulate multivalent delivery platforms represent a promising approach to surmount the aforementioned obstacles [10]. Formulation of antigens in particles in the viral or bacterial size range offers some attractive features, including protection of the antigen against degradation, facilitated uptake by antigen-presenting cells (APCs) through passive or active targeting, depot formation and co-delivery of antigens and adjuvants to the same APC, which could assist in directing the type of immune response desired [11]. Recently, viral nanoparticles (VNPs) have been an active area of research as delivery platforms for protein and peptide-based vaccines. Bacteriophage-derived nanoparticles; however, has attracted many attentions because of the unique advantageous features includinga good safety profile, intrinsic adjuvant properties, ease and cost-effectiveness of manufacturing. However, most widely used bacteriophage carriers have been derived from filamentous phages (M13 and f1) [12], [13], [14], [15]. The lytic T7 bacteriophage has the ability to display heterologous peptides (up to 40 amino acids) as a C-terminal fusion to all capsomers (415 copies) of 10B capsid protein or display larger proteins (up to 1200 amino acids) in mid-copy numbers (up to 15 copies) [16].T7 phage nanoparticles have been exploited previously for display of B cell epitopes such as Ep15 peptide of West Nile virus and an immuno-dominant region of Hepatitis B virus surface antigen (HBsAg) for diagnostic or vaccination purposes [17], [18]. In this study, we investigated immunogenicity and anti-tumor potential of chimeric T7 phage nanoparticles displaying anH-2k^d^-restricted CTL epitope (p66) derived from rat HER-2 in BALB/c mice. The T7-p66 phage nanoparticles effectively induced CTL responses in the absence of adjuvant. Moreover, this vaccine was shown to be effective in both prevention and treatment of a HER-2-positive breast tumor as demonstrated by successful rejection of implanted tumors and significant regression of established tumors in BALB/c mice.

## Materials and Methods

### Ethics Statement

All procedures used in this study were approved by the Institutional Ethical Committee and Research Advisory Committee of Tarbiat Modares University based on the National Specific Ethical Guidelines for Biomedical Research issued by Ministry of Health and Medicinal Education (MOHME) of Iran in 2005.

### Mice and Cell Line

Female 6–8 weeks old BALB/c mice (H-2^d^ haplotype) were purchased from the Pasteur Institute of Iran (Karaj, Iran) and were allowed to acclimate to our animal facility for one week before starting the experiments. The mice were maintained on a 12-h light/12-h dark cycle and received food and water *ad libitum*.

TUBO (Turin-Bologna) is a cloned cell line generated from a spontaneous mammary gland tumor from BALB-neuT transgenic mice and over-expresses HER-2 protein on the cell membrane [19]. This cell line was a generous gift from professor Pier-Luigi Lollini, University of Bologna, Italy. Although, rat HER-2 protein is a xenogeneic protein in normal mice (6% of the amino residues differ from the mouse ErbB2), TUBO cells do not appear to induce antibodies or any detectable CTL when implanted in wild-type BALB/c mice [19]. The cells were cultured in DMEM containing 20% FCS, 100 U/ml penicillin and 100 µg/ml streptomycin. Expression of the rat HER-2 protein in TUBO cells was confirmed by RT-PCR using the primers Forward: 5′-ATTCATCATTGCAACTGTAGA-3′ and Reverse: 5′-AAGCACCTTCACCTTCCTTA-3′.

### Peptides and Primers Synthesis

The 9-mer peptide p66 (TYVPANASL) corresponding to H-2K^d^-restricted, dominant CTL epitope from rat HER2/neu protein was used for mice immunizations, *in vitro* stimulation of splenocytes in IFN-γ ELISPOT and preparation of target cells for cytotoxicity analysis. This peptide consists of amino acid residues 66–74 and previously has been shown to be a dominant CTL epitope of rat HER-2 in BALB/c mice [20]. The p66 peptide and a di-epitope (p66x2) comprising two copies of p66 peptide with alanine-alanine (AA) flanking residues and a C-terminal FLAG epitope (**AA**TYVPANASL**AA**TYVPANASL**AA** *DYKDDDDK*) were synthesized and formulated with Freund's adjuvant as the equivalent peptide vaccines (FLAG epitope is shown in italics). A synthetic peptide (SYVPSAEQI) corresponding to an H-2K^d^-restricted CTL epitope from *Plasmodium yoelii* cicumsporozoite protein (PyCSP) was utilized in ELISPOT and cytotoxicity assays as an irrelevant control peptide [21]. The purity (>95%) and identity of peptides were determined by analytic high-performance liquid chromatography (HPLC) and mass spectrometry analysis (GenScript, USA). All primers used in sequencing and cloning steps were synthesized by Eurofins MWG, Germany. The primer sequences are described where they are used.

### Design and Synthesis of p66 and p66x2 DNA Inserts

The p66 and p66x2 peptide sequences were back translated in a DNA coding strand and codon optimized using GENEius software (Eurofins WMG, Germany) according to the codon usage table described for *Escherichia coli* strain *B* in Codon Usage Database (http://www.kazusa.or.jp/codon/). Single-stranded overhangs corresponding to EcoRI (5′-AATT-3′) and HindIII (5′-AGCT-3′) restriction sites were added at the 5′-end of sense and anti-sense strands respectively to allow directional cloning of the annealed DNA inserts in EcoRI/HindIII double digested and dephosphorylated genomic arms of T7Select415-1b phage vector (Novagen, USA).The DNA strands for p66 peptide were synthesized with 5′-phosporylations (Generay Biotech, Shanghai, China) as below:

(Sense: 5′-**AATT**CGGGCGGCGGCAGCACCTATGTGCCGGCGAATGCGAGCCTG**TAA**-3′) and antisense: 5′-**AGCT**CAGGCTCGCATTCGCCGGCACATAGGTGCTGCCGCCGCCCG-3′). A glycine-glycine-glycine-serine (GGGS) linker peptide was engineered at the DNA level to create a flexible spacer between p66 or p66x2 and the 10B capsid protein of the recombinant T7 phage. As shown in bold type, a TAA stop codon was inserted downstream of the p66-coding sequence to avoid any C-terminus extension of the p66 peptide by the amino acids encoded by the downstream restriction sites.

The synthetic DNA encoding p66x2 peptide (*GGGS* **AA**TYVPANASL**AA**TYVPANASL**AA** *DYKDDDDK*) was first cloned into pcDNA3.1(-) plasmid (Generay Biotech, Shanghai, China), which served as a template for amplification by a high-fidelity PCR using *pfu* DNA polymerase (Fermentas), pcDNA3.1-p66x2 template and the plasmid backbone primers (Forward: 5′-TAGCGGTTTGACTCACGG-3′) and (Reverse: 5′-ATGCCTGCTATTGTCTTCC-3′). The PCR product was digested with EcoRI and HindIII restriction enzymes, separated on a 3% agarose gel and the p66x2-endoding 130 bp insert was purified using QIAquick Gel Extraction Kit (QIAGEN).

### Construction of T7-p66 and T7-p66x2 Chimeric Phage Nanoparticles

**Figure 1:**
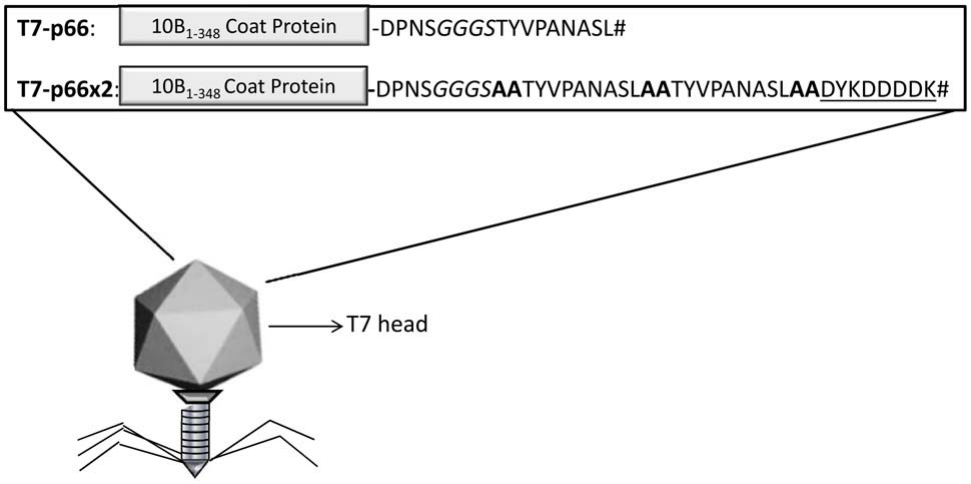
A schematic diagram depicting 10B capsomer structure in T7-p66 and T7-p66x2 phage nanoparticles. In T7Select415-1b vector, T7 phage capsid protein (10B) is composed of amino acid residues 1–348 followed by a multiple cloning site. In T7-p66, the rat HER-2 p66 CTL epitope (TYVPANASL) was fused to 10B protein using a GGGS linker. A dimer of p66 epitope with alanine-alanine flanking residues (bold) and a C-terminal FLAG (underlined) was cloned into 10B capsid protein to construct T7-p66x2 phage nanoparticles. TAA stop codon (#) was designed at the end of the corresponding oligonucleotides to avoid any C-terminal extension of the displayed epitopes by multiple cloning site-encoded amino acids.

The T7Select415-1b cloning kit containing the T7Select415-1b EcoRI/HindIII double-digested and dephosphorylated T7 phage genomic arms (Novagen, USA) was used to display p66 and p66x2 peptides on the T7 phage head as a fusion to the C-terminus of 10B capsid protein (Fig. 1). The p66x2 peptide was designed and displayed as a model to evaluate cross-presentation potential of polytope-displaying T7 phage nanoparticles for anti-tumor CTL induction. The p66-encoding insert was prepared by annealing of the aforementioned synthetic oligonucleotides. Briefly, an equimolar concentration of the two strands were mixed, heated in 95°C water and allowed cool slowly to room temperature (RT). Two microliters of the annealed oligonocleotide or 0.5 µg of the p66x2 insert (prepared by EcoRI/HindIII double digestion of pcDNA3.1-p66x2 PCR product) was then ligated to 0.02 pmol of EcoRI/HindIII digested and dephosphorylated T7Select415-1b vector arms (Novagen, USA). The ligation reaction was performed after addition of 1 µl T4 ligase (Fermentas) in a final volume of 5 µl and incubation at 16°C for 16 h. Before performing *in vitro* packaging, the ligation reactions were verified by PCR using primers provided by the manufacturer (Up primer: 5′GGAGCTGTCGTATTCCAGTC-3′ and Down primer: 5′-AACCCCTCAAGACCCGTTTA-3′). The PCR reaction was performed for 35 cycles in a thermocycler (Eppendorf, Germany) followed by electrophoresis on a 3% agarose gel and staining with SYBR® Green fluorescent dye.

### *In vitro* Packaging of T7-p66 and T7-p66x2 Chimeric Phage Nanoparticles

The T7 phage head contains 415 copies of 10B capsid protein, thus p66 and p66x2 peptides are displayed on the resulting T7 nanoparticles with a high copy number. To encapsidate recombinant T7 phage genomes, 4 microliters of the ligation reaction was mixed with 25 µl of T7 phage packaging extract (Novagen, USA) and incubated for 2 h at room temperature. Full-length T7 phage genomic DNA (without insert) was also packaged and used as control phage nanoparticles (T7-wt) in all *in vitro* and *in vivo* experiments. The packaging reactions were stopped by adding 270 µl of sterile Luria-Bertani (LB) broth and the packaging efficiency was evaluated after phage titration by plaque assay.

### Quantification of T7 Phage Nanoparticles by Plaque Assay

*In vitro* packaged T7-p66, T7-p66x2 and T7-wt nanoparticles were quantified by plaque assay as recommended by the manufacturer (Novagen, USA). In brief, 100 µl of serial log_10_ dilutions of the T7 nanoparticles in sterile LB broth was mixed with 250 µl of an overnight culture of *E.coli* BL21 (OD_600_ = 1) (Novagen, USA) and plated on LB agar plates after addition of 3 ml top agarose (1% Bacto-tryptone, 0.5% yeast extract, 0.5% NaCl, 0.6% agarose). The plates were incubatedat 37°C until complete development of plaques (3–4 h). The plaques were counted and the titers reported in plaque forming unit (PFU)/ml.

### Verification of Recombinant Plaques by DNA Sequencing and Plaque Lift Analysis

A portion of the top agarose of several well isolated plaques was scraped up using a sterile Pasteur pipette tip and dispersed in 100 µl of 10 mM EDTA, pH 8.0.The tube was heated for 10 min at 65°C and clarified by centrifugation at 14,000 g for 3 min. PCR reaction was performed on 2 µl of the clarified sample using *Taq* DNA polymerase (Fermentas) and screening for T7-p66 clones was performed by sequencing using T7 UP and Down primers. To verify T7-p66x2 plaques, a plaque lift assay was performed followed by immunoscreening for FLAG epitope, according to the protocol described for T4 phage by Jinag *et al.* with some modifications. Briefly, plaques resulting from T7-p66x2 or T7-wt packaging reactions were transferred onto nitrocellulose membranes for 40 min at 4°C, air-dried for 30 min and blocked with skim milk (5% in TBS) for 60 min. The membranes were washed once in TBS containing 0.05% Tween-20 (TBST) and incubated with 1/1000 dilution of anti-FLAG monoclonal antibody (Abcam) in TBS plus 0.1% BSA for 2 hours at RT. The bound antibodies were detected by an alkaline phosphatase (AP)-conjugated goat anti-mouse IgG (Abcam) and subsequent addition of a chromogenic mixture of 5-Bromo-4-Chloro-3 Indolylphosphate p-Toluidine salt (BCIP) and Nitro Blue Tetrazolium (NBT) (Novagen, USA). After development of the stained plaques (2–3 min), the membranes were rinsed with deionized water and dried at room temperature. Positive FLAG-displaying plaques were picked by aligning the membrane with plates and amplified as described below.

### SDS-PAGE and Western Blot Analysis of Chimeric T7 Phage Nanoparticles

The SDS-PAGE and western blot analysis were performed according to the protocol described by Hashemi *et al.* (manuscript submitted for publication). Briefly, T7-p66, T7-p66x2 and T7-wt phage nanoparticles (as negative control) were analyzed on a 12% polyacrylamide gel followed by Coomassie brilliant blue staining. For western blotting, proteins were transferred onto nitrocellulose membranes (Sigma) along with pre-stained protein markers using a semi-dry blotting device (APELEX, France) for 2 hours. Membranes were blocked with 5% skim milk in TBS overnight at 4°C. The chimeric 10B-p66x2 protein (∼40.66 kDa) was detected after addition of anti-FLAG mAb (1/1000 dilution) followed by extensive washing with TBST. Next, the membrane was incubated with AP-conjugated goat anti-mouse IgG polyclonal antibody (Abcam) for 60 min and the protein bands were visualized by adding a mixture of BCIP/NBT (Novagen). Considering the small size of p66 peptide (∼1 kDa), DNA sequencing was used to verify T7-p66 plaques expressing 10B-p66 chimeric capsid protein.

### Analysis of Surface Accessibility of p66x2 Peptide by Phage ELISA

Surface accessibility of the p66x2 peptide on the surface of T7 nanoparticles was assessed by phage ELISA. Ninety six-well high binding ELISA microplates (COSTAR, USA) were coated with T7-p66x2 or T7-wt phage nanoparticles (10^9^ PFU) in 100 µl of Tris-NaCl buffer (1 M NaCl, 10 mM Tris-HCl, pH 8.0) or the synthetic p66x2 peptide (10 µg/ml) in carbonate-bicarbonate buffer (100 mM, PH 9.6) overnight at 4°C. Plates were blocked with 1% BSA in PBS for 90 minat 37°C and washed once with PBST. Anti-FLAG mAb (Abcam) was added to wells at 1/2000 dilution in PBS plus 0.1% BSA. After incubation at 37°C for 60 min and performing washing steps, AP-conjugated goat polyclonal anti-mouse IgG (1/20,000 dilution) was added. A yellow color was developed after addition of SIGMA-FAST para-nitro phenyl phosphate (pNPP) substrate in Tris buffer which was stopped by addition of 50 µl of 3N NaOH. Absorbance of the plates was read at 405 nm using a microplate ELISA reader (TECAN). All samples were tested in triplicate.

### Large-scale Amplification and Purification of T7 Phage Nanoparticles

The T7-p66, T7-p66x2 and T7-wt nanoparticles were propagated in the log phase culture of *E. coli* BL21 (OD_600_∼0.8) grown in M9-LB broth (LB broth supplemented with 50 ml 20X M9 salts, 20 ml 20% glucose and 1 ml of 1 M MgSO_4_ per liter) at a multiplicity of infection (MOI) of 0.001 and incubated at 37°C until complete lysis of the culture (3–6 hours). Thirty minutes before removing the culture from the shaker, DNAse I and RNAse A (Roche, Germany) were added to degrade released bacterial nucleic acids. T7 phage nanoparticles were precipitated from the culture supernatant by addition of 1 M NaCl and 10% polyethylene glycol (PEG 6000, Merck) followed by overnight incubation at 4°C. The T7 phage pellet was resuspended in Tris-NaCl buffer (PH 8) and PEG and cell debris was removed by centrifugation at 10,000 rpm for 10 min. To remove residual PEG and debris, an equal volume of chloroform was added, gently inverted and the top aqueous phase was harvested after low-speed centrifugation at 4°C. The purified T7 nanoparticles were sterilized using a pyrogen-free 0.2 µm pore-size cellulose acetate filter (Millipore) and stored at 4°C until further analysis.

### Removal of Bacterial Endotoxin from T7 Phage Nanoparticles

The bacterial endotoxin (LPS) concentration in all T7 nanoparticle preparations was determined in triplicate using a sensitive colorimetric Limulus Amebocyte Lysate (LAL) QCL-1000® kit (Lonza, USA) according to the manufacturer's instructions. LPS was removed from T7 phage nanoparticles based on a method for removal of endotoxin from protein solutions by phase separation using Triton X-114 as described by Aida *et al*. [22] and modified by Hashemi *et al*. (manuscript submitted for publication).

### Immunization Schedule

Six-eight weeks old female BALB/c mice were randomly divided into 8 groups as shown in table 1 (n = 6/each group, 8 groups) and received three subcutaneous injections on days 0, 14 and 28. Each mouse was administered with 10^10^pfu of T7-p66 or T7-p66x2 phage nanoparticles. To verify whether the multivalent display of p66 peptide on T7 nanoparticles was necessary for induction of specific CTL responses, 50 µg of the p66 peptide and 10^10^pfu of T7-wt phage nanoparticles were mixed and co-injected into mice. Control groups of mice received 10^10^pfu of T7-wt nanoparticles, Freund's adjuvant (CFA/IFA) or PBS. Fifty micrograms of the p66 and p66x2 peptides was emulsified 1∶1 in complete Freund's adjuvant (CFA) for priming and in incomplete Freund's adjuvant (IFA) for booster injections.

**Table 1:** Immunization groups.

Group number	Group	Vaccine formulation
I	T7-p66	10^10^ PFU of T7-p66 nanoparticles
II	T7-p66x2	10^10^ PFU of T7-p66x2 nanoparticles
III	p66+FA	p66 peptide emulsified 1∶1 in CFA/IFA
IV	p66x2+FA	p66x2 peptide emulsified 1∶1 in CFA/IFA
V	T7-wt+p66	A mixture of p66 peptide (50 µg) and T7-wt phage (10^10^ PFU)
VI	T7-wt	10^10^ PFU of T7-wt nanoparticles
VII	CFA/IFA	Emulsion of PBS with CFA in priming and IFA in boosters
VIII	PBS	PBS buffer

### Splenocyte Culture

Mice were inoculated subcutaneously three times at 2-week intervals. Seven days after the third immunization, mice were sacrificed by cervical dislocation and spleens were aseptically removed and homogenized in complete RPMI-1640 medium (10% FBS, 100U/ml penicillin, 100 µg/ml streptomycin, 2 mM L-glutamine and 1 mM sodium pyruvate and HEPES buffer). Red blood cells (RBCs) were lysed using Tris-ammonium chloride lysis buffer (0.16 M NH_4_Cl and 0.17 M Tris-HCl) after removal of debris by filtration through a 70 µm nylon cell strainer (BD, USA). Finally splenocytes were resuspended in complete RPMI-1640 medium and cell viability was determined by trypan blue dye (0.4% w/v) exclusion. All cell culture reagents were purchased from Gibco® (Invitrogen, USA) unless otherwise specified.

### *Ex vivo* Analysis of p66-specific T cell Responses by Interferon-γ (IFN-γ) ELISPOT and IL-4 ELISA

The p66 peptide-specific T cell responses were evaluated using a mouse IFN-γ Enzyme-linked Immunosorbent Spot (ELISPOT) assay (eBioscience, USA) following the manufacturer's instructions. Briefly, on day 7 after the last injection, 96-well Multiscreen IP Plates (Millipore, USA) were coated with 100 µl of assay diluent containing anti–IFN-γ monoclonal capture Ab and incubated overnight at 4°C. After blocking the plate with complete RPMI-1640 medium at room temperature for 60–90 min and washing steps, splenocytes depleted of RBCs were added to wells (300,000 cells/well in 100 µl) in triplicate and stimulated with p66 Peptide (5 µg/ml).PHA (4 µg/ml) (Sigma, USA) served as a positive control and *Plasmodium yoelii* H-2K^d^ peptide (as irrelevant peptide: SYVPSAEQI) was used as a negative control (5 µg/ml). The plate was incubated for 36 hours at 37°C/5% CO2 and washed with PBST buffer. Next, a biotinylated anti-mouse IFN-γ detection antibody was added and incubated for 2 hours at RT. Unbound detection antibody was washed, the enzyme conjugate (Streptavidin-HRP) was added and incubated for 45 min at RT. Spots were developed on the membranes after addition of a mixture of aminoethylcarbazole (AEC) (Sigma) substrate and H_2_O_2_ in acetate buffer. The plates were thoroughly washed and allowed to air dry overnight, and spot forming cells (SFCs) were counted under a dissecting microscope (Nikon, USA).

For measurement of Interleukin 4 (IL-4) secretion as a prototypical Th_2_ cytokine, splenocytes were plated in 24-well plates (5x10^6^ cells/2 ml/well) and stimulated *in vitro* with p66 peptide (10 µg/ml) or T7-wt phage nanoparticles (10^9^ PFU/ml) in the presence of recombinant mouse IL-2 (15U/ml) (Roche, Germany) for 72 hours at 37°C and 5% CO2. The endotoxin-free T7-wt nanoparticles were used for *in vitro* stimulation of the splenocytes to assess anti-T7 capsid T cell responses. The irrelevant H-2K^d^-restricted epitope from *Plasmodium yoelii* (10 µg/ml) and PHA (4 µg/ml) were used as negative and positive controls respectively. The culture supernatants were harvested by centrifugation at 4°C and IL-4 was measured using a mouse IL-4 commercial kit (R&D, USA) according to the protocol recommended by the manufacturer and IL-4 concentrations were reported in pg/ml.

### Evaluation of Cytotoxic T lymphocytes by Lactate Dehydrogenase (LDH) Assay

The mice splenocytes harvested 7 days after the second booster injection, were stimulated *in vitro* with p66 peptide (10 µg/ml) and recombinant mouse IL-2 (15 U/ml) for 72 hours at 37°C and 5% CO2. Viable cells were counted and used as effector cells for the measurement of specific cytolytic activity in a standard lactate dehydrogenase (LDH) release assay. The P815 mastocytoma cells (H-2^d^) were pulsed with p66 peptide (10 µg/ml) for 2 h at 37°C and used as target cell. The target cells were distributed into triplicate wells of a 96-well plate (5×10^4^ cells/well) in 100 µl of RPMI-1640 without phenol red or serum but supplemented with 2% BSA. Next, the lymphocytes were serially diluted in the same medium and added to target cells in triplicate with (effector-to-target) E:T ratios of 100∶1, 50∶1 and 25∶1 in a total volume of 0.2 ml. Unpulsed P815 cells or pulsed with the irrelevant peptide were used as controls. Wells containing only target cells or splenocytes with RPMI-1640 medium only served as spontaneous release and P815 target cells were treated with 0.5% Triton X-100 to determine maximal release. The plates were incubated for 6 hours at 37°C and 5% CO2. The supernatant was collected and assayed for LDH following the manufacturer's instructions (Roche, Germany). Finally, absorbance of the water-soluble formazan was measured at 490 nm using an ELISA reader (TECAN). The percentage of specific lysis was calculated as follows: specific lysis (%) = 100 × (experimental release - spontaneous release)/(maximum release - spontaneous release).

### ELISA Characterization of Antisera forT7 Capsid-specific IgG Isotypes

Seven days following the final immunization, mice sera were collected by bleeding through the retro-orbital plexus. The complement was heat-inactivated for 30 min at 56°C and the sera were stored at -20°C until analysis. Ninety-six well high binding ELISA microplates (COSTAR, USA) were coated with 10^9^pfu of T7-wt phage nanoparticles in Tris-HCl buffer (1 M NaCl, 10 mM Tris-HCl, pH 8.0) overnight at 4°C. The plates were washed once with PBST buffer and blocked with PBS containing 1% BSA (Merck, Germany) for 2 hours at 37°C. Mice sera were serially diluted in PBS/0.1% BSA, added to wells in triplicate and incubated for 60 min at 37°C. After washing with PBST, AP-conjugated goat anti-mouse antibodies were added including anti-mouse IgG (1/20000, Abcam), anti-mouse IgG_1_ (1/2500, Abcam) and anti-mouse IgG_2a_ (1/2500, Abcam) and incubated for 90 min at RT. Finally, SIGMA-FAST para-nitro phenyl phosphate (pNPP) substrate in Tris buffer (Sigma) was added and the reaction was stopped by adding 50 µl of 3N NaOH. Absorbance of the plates was read at 405 nm using a microplate ELISA reader (TECAN).

### Prophylactic Model of TUBO Tumor Challenge

In the prophylactic setting, six mice per group were vaccinated as described above. Seven days after the second booster injection, mice were anesthetized with a mixture of 10% ketamine (100 mg/kg) and 2% xylazine (10 mg/kg) and challenged subcutaneously at the opposite flank with 5x10^5^ TUBO cells in 100 µl sterile PBS. Mice were monitored regularly twice a week and considered tumor-free until a non-regressing tumor larger than 1 mm^3^ was detected.

### Therapeutic Model of TUBO Tumor Challenge

In the therapeutic setting, mice were first challenge with 5x10^5^ TUBO cells in 100 µl PBS. When 100% of mice had an established tumor diameter of ∼3 mm in the greatest dimension, immunization of mice was initiated as described. Each mouse was monitored twice a week for tumor growth which was measured in two perpendicular diameters using a caliper. Tumor volume (mm^3^) was calculated using the formula *V = a × b^2^/2*, where "*a*" is the largest and *"b*" is the smallest diameter and represented as mean volume of tumors in each group. Mice were euthanized if the tumor diameter reached 15 mm or it was ulcerated or if animals showed signs of cachexia.

### Statistical Analyses

The significance of the differences among various groups was determined with One-way analysis of variance (ANOVA) followed by Tukey's post-test. Data were expressed as means ± standard deviation (SD). Differences were considered statistically significant when P<0.05. Survival data, expressed as percent tumor-free mice, was analyzed by Kaplan–Meier method and log-rank test was used to compare survival curves between groups. All statistical analyses were performed using GraphPad Prism 5 Software (San Diego, USA).

## Results

### The p66x2 Peptide is Efficiently Displayed on T7-phage Capsids

**Figure 2:**
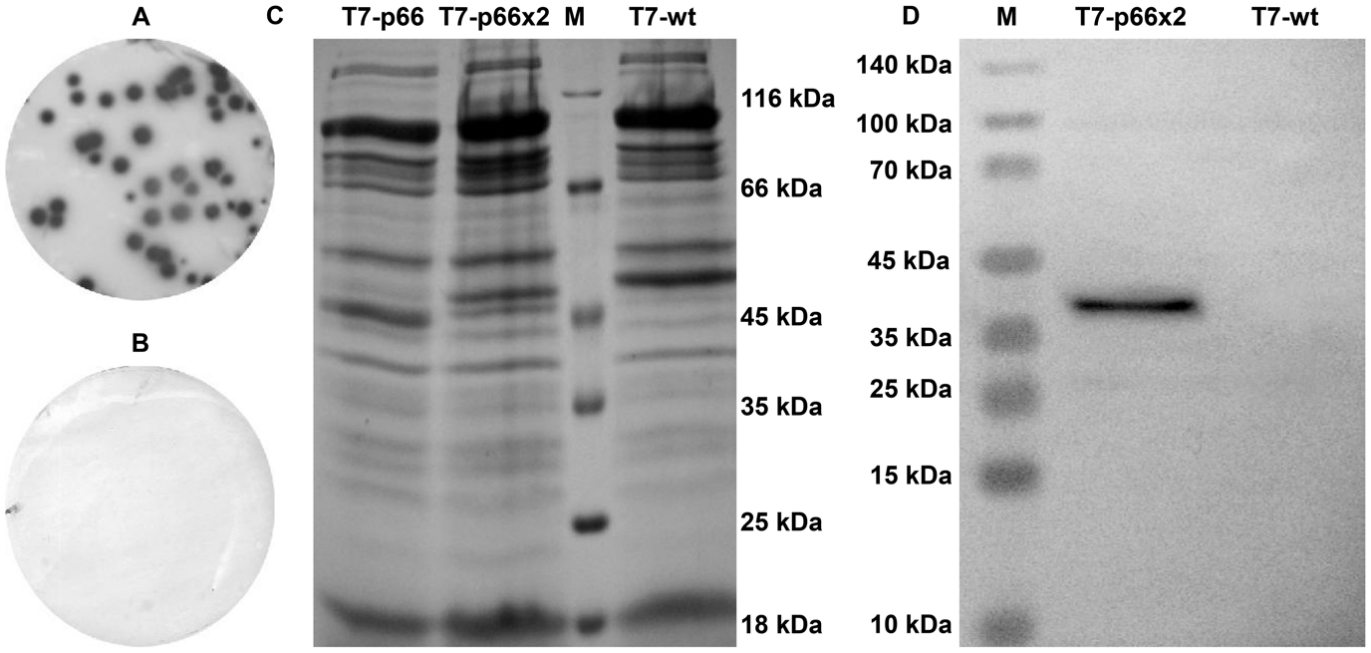
Plaque lift assay and characterization of chimeric T7 phages. The T7-p66x2 (A) or T7-wt plaques (B) developed after plating of *in vitro* packaging reactions were transferred onto nitrocellulose membranes. An anti-FLAG monoclonal antibody was used to detect recombinant phages blotted onto membrane followed by addition of an AP-conjugated secondary antibody and a substrate mixture of BCIP/NBT. As shown, the p66x2 peptide is easily accessible to monoclonal antibodies (A) and no reactivity is observed on the membranes blotted by T7-wt plaques as negative control (B). Coomassie blue-stained SDS-PAGE electrophoresis (C). Lane 1: T7-p66; lane 2: T7-p66x2; lane 3: protein marker and lane 4: T7-wt. (D) Western blot analysis using the anti-FLAG monoclonal antibody and anti-mouse IgG AP-conjugate. Lane 1: Pre-stained protein markers; Lane 2∶10B-p66x2 chimeric protein with a molecular weight of ∼40.66 kDa; Lane 3: T7-wt as negative control. No degradation product of 10B-p66x2 protein is observed as only one band was detected. Non-specific reaction of the antibodies to T7 capsid proteins was not observed.

Plaques developed after *in vitro* packaging reaction were transferred onto nitrocellulose membranes and detected using anti-FLAG antibody followed by an AP-conjugated polyclonal anti-mouse IgG. As shown in Figure 2, when fused to all 415 copies of 10B capsomers, the p66x2 peptide was efficiently displayed on the surface of T7 phage nanoparticles without interfering with cytoplasmic packaging of the infectious capsids or the integrity of the nanoparticles. Stability is an important pre-requisite for a vaccine. To address this issue, both T7-p66x2 and p66x2 nanoparticles were shown to resist against harsh denaturing conditions including 10 mM EDTA and 1% SDS at 37°C, similar to what has been described for T7-wt phage nanoparticles [16].

### SDS-PAGE and Western Blotting

The expression and sizes of chimeric 10B capsomers (10B-p66 and 10B-p66x2) were further determined by SDS-PAGE and WB as described. A single protein band of ∼ 40.66 kDa MW was detected in T7-p66x2 lane as predicted by *in silico* calculation using EnCor Biotechnology Inc. online software. The corresponding protein band was not visible in the wild-type T7 (T7-wt) nanoparticles as negative control (Fig. 2).

### Phage ELISA Confirmed Surface Accessibility of p66x2 to mAb

To investigate the surface accessibility of the p66x2 peptide to specific antibodies when displayed on T7 phage nanoparticles, the purified T7-p66x2 along with T7-wt phage nanoparticles and synthetic p66x2 peptide were coated onto a 96-well microplate followed by detection with anti-FLAG mAb and AP-conjugated anti-mouse IgG as described. An intense yellow color was developed in wells coated with T7-p66x2 nanoparticles (10^9^pfu) comparable to those coated with p66x2 peptide (10 µg/ml) indicating strong reactivity of the displayed peptide with mAb. No color was detected in wells coated with T7-wt phage nanoparticles, demonstrating no binding of the anti-FLAG mAb to the backbone of the non-chimeric T7 phage capsids (data not shown).

### Contaminating LPS was Effectively Removed by Triton X-114 Phase Separation

The method used for LPS removal from all T7 phage nanoparticles in this study, successfully reduced LPS concentration. However, depending on the T7 phage preparation, up to five cycles of Triton X-114 extraction were required to achieve LPS concentration in the detection limit of the QCL-1000 LAL kit (0.1–1EU/ml). The LPS levels for all T7 preparations were between 0.2–0.8 EU/ml.

### T7-p66 Chimeric Nanoparticles Induce a Higher Frequency of IFN-γ-secreting T cells

**Figure 3:**
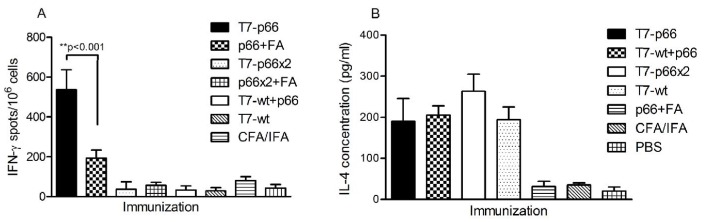
Analysis of p66 peptide- and T7 capsid-specific T cell responses. (A) Evaluation of p66-specific T cell responses by IFN-γ ELISPOT. One week after the last boost, mice were sacrificed and their spleens harvested. P66 peptide-specific response was measured by IFN-γ ELISPOT after subtracting background IFN-γ-secreting T cells in the presence of the irrelevant control peptide. The mean and standard deviation (SD) of each group is shown. Analysis of the results by one-way ANOVA followed by Tukey's post-test showed a significantly higher frequency of IFN-γ-secreting T cells in the mice immunized with endotoxin-free T7-p66 phage nanoparticles compared to those inoculated with p66 peptide (50 µg) emulsified in Freund's adjuvant (p<0.001); even though about 1000-fold less amount of p66 peptide (50ng) was carried by the T7-66 nanoparticles inoculated (10^10^pfu/mouse). As shown, T7-p66x2 nanoparticles or an emulsion of p66x2 peptide in Freund's adjuvant failed to induce a significant IFN-γ response compared to negative controls injected with T7-wt phage or CFA/IFA. (B) T7-phage nanoparticles are able to drive IL-4 secretion in T cells. Splenocytes from the immunized mice were stimulated *in vitro* with p66 peptide, the irrelevant peptide or T7-wt phage nanoparticles in the presence of recombinant mouse IL-2. After 72 hours, the culture supernatants were harvested and IL-4 concentration was measured by ELISA in triplicate. As depicted, significant levels of IL-4 were released by the splenocytes of the mice immunized with various formulations of either wild-type or recombinant T7 phage nanoparticles compared to those not receiving T7 phages. However, there was no significant difference between T7-wt and various chimeric nanoparticles.

Mouse IFN-γ ELISPOT assay was performed to evaluate specific T cell responses against the displayed p66 CTL epitope *ex vivo* (Fig. 3A). Seven days after the last booster injection, mice splenocytes were isolated and stimulated with p66 peptide for 36 hours as described above. A significant difference was observed in the number of spot-forming T cells (SFC) in response to stimulation with p66 peptide in the mice immunized with T7-p66 phage nanoparticles compared to the negative controls including T7-wt, CFA/IFA and PBS groups. Interestingly, endotoxin-free T7-p66 phage nanoparticles provoked a significantly higher IFN-γ response compared to the animals injected with 50 µg of the synthetic p66 peptide emulsified in CFA/IFA (p<0.001); even though the inoculated dose of T7-p66 (10^10^pfu/mouse) carried only about 50 ng of p66 peptide (∼1000-fold lower dose) as calculated *in silico* (Fig. 3A).

When stimulated with p66 peptide, the splenocytes from the mice immunized with a mixture of T7-wt and p66 peptide did not show a significant frequency of p66-specific IFN-γ spots compared to those reactivated with the irrelevant peptide or media only. Surprisingly, IFN-γ ELISPOT of T7-p66x2 group or those injected with p66x2 peptide emulsified in CFA/IFA adjuvant (p66x2+FA group) had no significant differences compared with mock groups or those cultured un-stimulated (media only) or stimulated with the irrelevant peptide. These data indicate that only T7-p66 phage nanoparticles carrying a single copy of p66 peptide were successfully cross-presented and elicited a specific T cell response against the displayed CTL epitope.

### T7-phage Nanoparticles Increase Secretion of IL-4 Cytokine

Seven days after the last immunization, splenocytes were cultured and stimulated with p66 peptide, endotoxin-free T7-wt nanoparticles or the irrelevant peptide for 72 hours. The culture supernatants were collected and assayed for Th_2_ cytokine IL-4 by ELISA (Fig. 3B). The p66 peptide stimulation did not induce any significant amounts of IL-4 compared to un-stimulated or the irrelevant peptide-stimulated splenocytes (data not shown). Indeed, the p66 peptide could not function as a T helper epitope. However, when stimulated with T7-wt phage nanoparticles, splenocytes secreted significantly higher levels of IL-4 compared to negative controls inoculated with PBS or CFA/IFA (p<0.001). No significant difference was detected in IL-4 concentration in supernatants of the splenocytes from the mice immunized with T7-p66, T7-wt+p66, T7-p66x2 or T7-wt phage nanoparticles indicating that the displayed or co-administered p66 peptide did not contribute to or interfered with induction of T helper responses against T7 capsid proteins.

### Robust IgG Antibody Responses are Elicited against the T7 Phage Capsid

**Figure 4:**
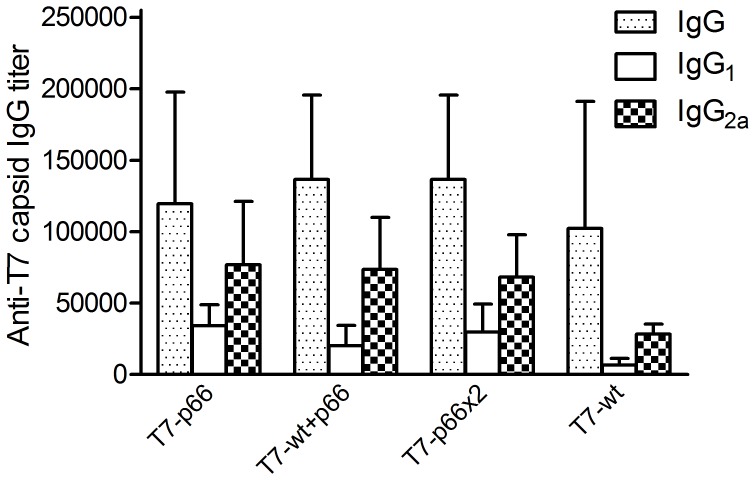
Robust IgG antibody responses are induced against T7 phage capsids. Blood samples were collected through the retro-orbital plexus on day 7 after the last booster injection and total IgG and IgG_1_/IgG_2a_ subclass antibody titers were determined using an ELISA. The data represent the mean IgG titers in 5 mice ± standard deviation indicated by error bars. PBS-inoculated animals had a background IgG titers (not shown). The end-point titer of each sample was determined as the highest dilution that yielded an OD_405nm_ value greater than twice that of similarly diluted serum sample collected pre-vaccination. As shown, all mice vaccinated with various formulations of T7 phage nanoparticles produced a high titer of anti-T7 capsid IgG. More importantly, IgG_2a_, a prototypical Th_1_-induced isotype, had a higher titer compared to IgG_1_, a Th_2_-induced isotype.

Seven days after the last booster injection, mice were bled through retro-orbital plexus and sera were collected and analyzed for IgG antibodies against T7 phage capsids (Fig. 4). Pre-existing antibodies against vaccine carrier proteins has been frequently reported to inhibit humoral immune response against antigens conjugated to the same carrier by a process termed carrier induced epitopic suppression (CIES) [23], [24]. To our knowledge, no study on CIES phenomenon has been reported when a CTL epitope is genetically fused or conjugated to a particulate carrier. To address this question, anti-T7 capsid IgG, IgG_1_ and IgG_2a_ antibodies were measured by serum ELISA on the microplates coated with T7-wt nanoparticles as described before. A significant level of IgG antibodies was elevated against T7 capsids in all T7 nanoparticle-immunized mice; however, no significant difference was observed between different T7 phage formulations. No detectable IgG was observed in sera from the mice vaccinated with CFA/IFA or PBS. Although both IgG_1_ and IgG_2a_ isotypes were generated against T7 capsids but IgG_2a_ amounts, a Th_1_-biased IgG isotype, were higher than IgG_1_, a Th_2_-biased isotype. These data support the idea that T7 phage platform predominantly activates a Th_1_ cell response as has been reported recently for a λ phage-based peptide and gene delivery system [25].

### Immunization with T7-p66 Chimeric Nanoparticles Induces CTL-mediated Lysis of Target Cells *in vitro*

**Figure 5:**
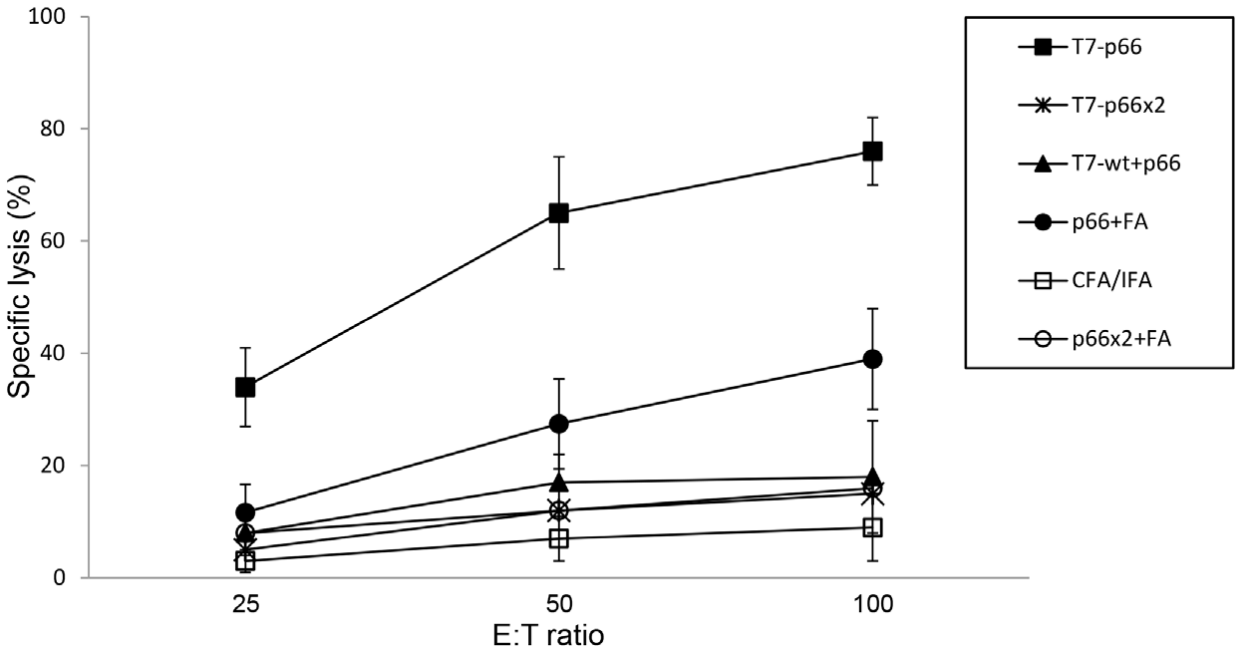
Cytotoxicity analysis by LDH assay. CTL activity of vaccinated BALB/c mice against P815 target cells loaded with synthetic p66 peptide was measured using LDH assay. The splenocytes were stimulated *in vitro* for 72 hours with p66 peptide, the irrelevant peptide or left un-stimulated and co-cultured with p66-pulsed p815 target cells at different E:T ratios for 6 hours. The effector cells lytic activity was measured as described in Materials and Methods. All data are representative of three independent experiments using pooled spleen cells from five mice and error bars (SD) were calculated based on triplicates. Splenocytes from the mice vaccinated with T7-p66 phage nanoparticles showed a significantly greater cytotoxicity against p66-loaded target cells than the mice receiving p66 emulsified in Freund's adjuvant (p66+FA group). Injection of mice with T7-p66x2 nanoparticles or an emulsion of p66x2 peptide in Freund's adjuvant did not result in a significant cytotoxic activity against target cells compared to negative controls (T7-wt, PBS and CFA/IFA). Similarly, a mixture of T7-wt nanoparticles and p66 peptide only induced a background level of cytotoxic activity in splenocytes similar to negative controls. Data corresponding to T7-wt and PBS have not been shown.

To demonstrate that immunization with chimeric T7-p66 phage nanoparticles induces peptide-specific CTLs capable of killing p66 peptide-pulsed target cells *in vitro*; the p66-stimulated splenocytes were co-cultured as effector cells with p66-pulsed P815 target cells at three different E:T ratios (25∶1, 50∶1 and 100∶1) for 6 hours. The supernatants were harvested and analyzed by LDH release assay as mentioned above. The data depicted in Fig. 5 indicate that splenocytes isolated from the mice immunized with T7-p66 phage nanoparticles effectively lysed P815 target cells. This killing was specific because only a background level of lysis of P815 target cells pulsed with an irrelevant peptide was observed. Furthermore, the mice injected with T7-p66 nanoparticles had a significantly higher specific lysis compared to p66 peptide emulsified in CFA/IFA adjuvant. As expected from IFN-γ ELISPOT results, no significant lysis of target cells was observed in the mice vaccinated with T7-p66x2 nanoparticles or an emulsion of p66x2 peptide in CFA/IFA compared to controls (Figure 5). A simple mixture of p66 peptide and T7-wt phage nanoparticles (T7-wt+p66 group) failed to induce a peptide-specific T cell response in mice indicating strong dependence of p66 CTL peptide immunogenicity to repetitive arrangement on the surface of T7 phage nanoparticles.

### T7-p66-vaccinated Mice Efficiently Reject HER2-expressing TUBO Cells

**Figure 6:**
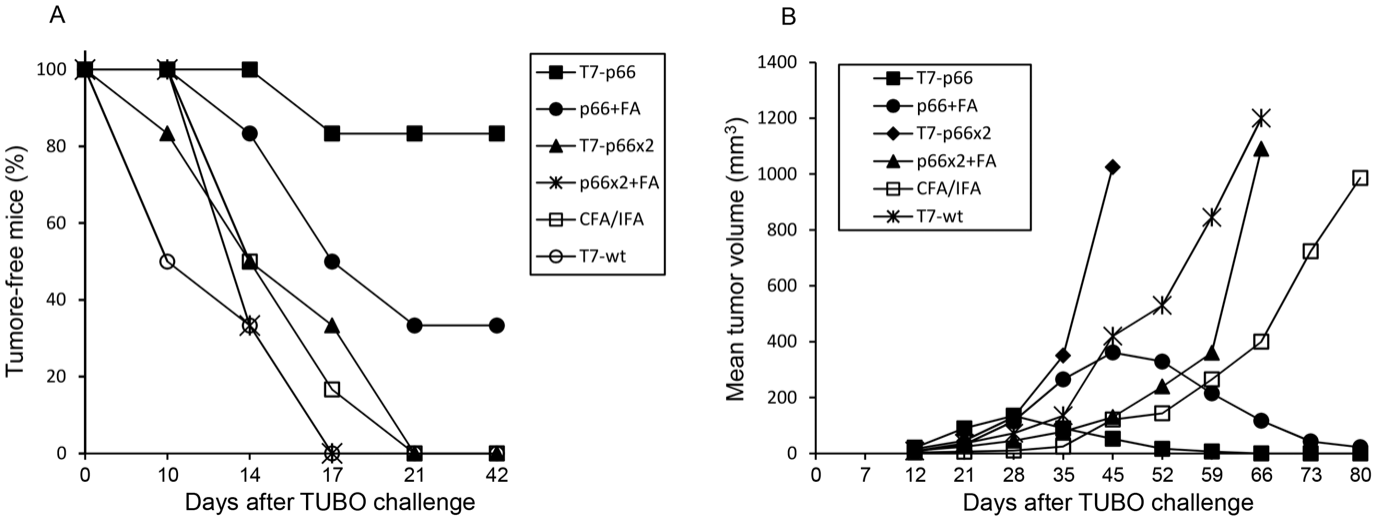
Anti-tumor effects of chimeric T7 phage nanoparticles. (A) Tumor incidence of the immunized mice in prophylactic setting. Seven days after the last boost, mice were challenged subcutaneously with 5×10^5^ TUBO breast cancer cells and monitored for palpable tumors for six weeks. As shown, 83% of mice (5 out of 6 mice) did not develop TUBO tumors and remained tumor-free until the end of study (day 42) (log-rank test, *P* = 0.004, compared to negative controls). In contrast, with p66 peptide-CFA/IFA emulsion only 33% survival rate was achieved (log-rank test, *P* = 0.04, compared to negative controls. All mice in T7-p66x2, p66x2+FA and T7-wt+p66 groups showed fast-growing tumors and had to be euthanized. (B) Therapeutic efficacy of the chimeric T7 phage nanoparticles. Before initiation of the immunization schedule, BALB/c mice were implanted with 5×10^5^ TUBO cells in the right flank. After developing palpable tumors of ∼3 mm in diameter, vaccination was started in the opposite flank and tumor sizes were recorded regularly as described. Immunization of mice with T7-p66 nanoparticles successfully resulted in regression of the established tumors in four out of six mice. The other two animals had ulcerated tumors and had to be euthanized by day 28. In contrast, only two out of six mice vaccinated with p66 peptide plus Freund's adjuvant (p66+FA group) was able to significantly control tumor growth by day 80; even though the tumors were not completely eradicated at the end of study. Furthermore, these mice had a greater mean tumor volume compared to T7-p66-injected animals throughout the monitoring period. All control mice as well as those vaccinated with T7-p66x2 or p66x2+FA succumbed to TUBO tumors.

To evaluate whether the CTL responses induced by p66 vaccination were potent enough to protect against HER-2-overexpressing tumors, BALB/c mice were vaccinated as described and 7 days later challenged subcutaneously with 5×10^5^ TUBO cells implanted in the opposite flank and monitored for tumor growth twice a week (Fig. 6A). A significant rejection of TUBO cells was observed in BALB/c mice immunized with T7-p66 nanoparticles. Interestingly, five out of six mice were remained tumor-free after TUBO cell challenge. The p66 peptide emulsified in CFA/IFA significantly showed a significantly lower protective effect against TUBO tumor challenge, as only two out of six mice remained tumor-free by day 42. In contrast, no protection against TUBO cell challenge was observed in control animals inoculated with T7-wt, CFA/IFA or PBS and all mice developed fast-growing tumors and had to been euthanized by week 3 post-challenge. In accordance with *ex vivo* results obtained in IFN-γ ELISPOT and cytotoxicity assay (LDH), immunization of mice with a mixture of T7-wt nanoparticles and p66 peptide failed to prevent TUBO tumor development in mice. Similarly, as depicted in Figure 6A, vaccination of mice with T7-p66x2 phage nanoparticles or p66x2 peptide-CFA/IFA emulsion did not result in a significant anti-tumor response as was expected from unsuccessful cross-presentation of the p66x2 di-epitope in the context of alanine-alanine spacers.

### Therapeutic Potential of T7-p66 Nanoparticles against Established HER-2-expressing Tumors

To examine anti-tumor potential of chimeric T7 phage nanoparticles against subcutaneously established tumors, mice were implanted with 5×10^5^ TUBO cells until palpable tumors (∼3 mm) were developed and then vaccinations were performed as described in section 2.12. As depicted in Figure 6B, 4 out of 6 mice immunized with T7-p66 phage nanoparticles had tumors that continued to grow for a few weeks but regressed completely by day 66 and remained tumor-free for 80 days. The remaining two animals had to be euthanized for humane reasons on day 28. Significant anti-tumor effects were obtained in the mice that were vaccinated with p66+FA and 2 of the 6 tumors regressed dramatically by day 80; however the other four animals had to be euthanized by day 45. In contrast, all control mice developed large tumors and had to be killed by day 80. However, some therapeutic advantage was observed in the mice that received CFA/IFA alone or emulsified with p66x2 peptide compared to PBS group. These mice had a less progressive tumor growth which would be attributed to the strong stimulatory effects of Freund's adjuvant especially CFA on the innate immunity because of several pathogen-associated molecular patterns (PAMPs) present in the heat-killed mycobacteria such as CpG motifs [26], [27]. Mice receiving a combination of T7-wt nanoparticles and p66 peptide (T7-wt+p66) or T7-p66x2 phage nanoparticles showed the same rate of survival as did negative controls and all were euthanized by the end of study.

## Discussion

Viral nanoparticles (VNPs) are attracting great interest for developing novel vaccines against infectious diseases and cancer. Particulate antigens like viruses have been proved to be delivered to APCs and cross-presented more efficiently than soluble antigens [28]. In recent years, nanoparticles derived from bacteriophages have attracted many attentions because of several important advantageous features including high multivalent display, intrinsic adjuvant activity, excellent safety profile and ease of manufacture. To date, there is a limited data on the immunogenicity and anti-tumor potential of bacteriophage nanoparticles displaying a TAA-derived CTL epitope *in vivo*. The filamentous fd phage virions displaying a peptide corresponding to the reverse transcriptase of HIV-1 have been shown to mount a specific CTL response in mice [29]. Similarly, Fang *et al*. demonstrated that fd phages displaying a CTL epitope from melanoma antigen (MAGE_161–169_) elicited CTL responses and showed both preventive and therapeutic effects against melanoma in mice [12]. Several TAAs were expressed on the surface of the T7 phage and shown to trigger specific immune responses in BALB/c mice following oral immunization. Furthermore, these immune responses inhibited tumor growth and metastasis of the 4T1 mammary adenocarcinoma cell line [30]. In another study, immunotherapy of mice with VEGFR2 displayed on T4 phage nanoparticles resulted in protective immunity against Lewis lung carcinoma (LLC) [31]. Immunization of mice with murine pneumotropic virus (MPtV) or murine polyomavirus (MPyV) VLPs carrying an ECD-TM (extracellular plus transmembrane domain) fragment of rat HER-2/neu was efficient both as a prophylactic and therapeutic tumor vaccine against rat HER-2-positive TUBO tumors [32], [33]. Jalali *et al*. demonstrated that vaccination with multi-epitope peptides from the rat HER2/neu encapsulated in liposome-polycation-DNA (LPD) nanoparticles induced an antigen-specific immunity and led to lower tumor sizes and longer survival time in TUBO tumor mice model [34]. It has been previously reported that co-administration of a dominant H2-K^d^-restricted CTL epitope of rat HER-2 (p66 peptide) with incomplete Freund’s adjuvant (IFA) and TLR9 agonist CpG ODN 1826 induced immune responses with prophylactic and therapeutic benefit against spontaneous mammary tumors in BALB-neuT transgenic mice [20]. In the present study, we investigated the immunogenicity and anti-tumor effects of chimeric T7 phage nanoparticles displaying 415 copy of p66 epitopeas a fusion to 10B capsid protein in an implantable breast cancer model in both prophylactic and therapeutic settings. BALB/c mice were immunized three times subcutaneously with 10^10^pfu of endotoxin-free T7-p66 phage nanoparticles. Controls received T7-wt, CFA/IFA adjuvant alone, or PBS. On day 35 (7 days after the last booster injection), single-cell suspensions of the splenocytes were re-stimulated *ex vivo* with p66 peptide and assayed for secretion of Th_1_ and Th_2_ cytokines. The T7 capsid elicited both humoral and cellular immune responses indicated by IgG_1_ and IgG_2a_ isotypes and secretion of Th_2_ cytokine IL-4 by T cells. However, predominantly Th_1_-biased IgG_2a_ subclass was generated against T7 capsids after immunization with all T7 phage nanoparticle formulations. The p66 peptide emulsified in Freund's adjuvant, a well-known inducer of Th_1_ cells, provoked peptide-specific T cell responses as demonstrated by significant *ex vivo* secretion of IFN-γ and specific cytotoxic activity against p66 peptide-loaded target cells. However, this group showed a significantly lower IFN-γ response and cytotoxicity compared to T7-p66-vaccinated mice; even though a 1000-fold higher concentration of p66 peptide (50 µg) was administered indicating the crucial role of repetitive display of p66 peptide on the surface of T7 nanoparticles in its immunogenicity. The results obtained by the immunization of mice with a mixture of T7-wt nanoparticles and p66 peptide (50 µg) further supported this idea where no effective T cell responses was detected against p66 peptide *in vitro* or implanted tumors *in vivo*. The CTL responses elicited by vaccination of mice with T7-p66 nanoparticles showed superior anti-tumor effects *in vivo* over p66 peptide-CFA/IFA emulsion indicated by a higher survival of mice (83% vs. 33%) against HER-2-expressing TUBO cell challenge and complete regression of the established tumors (66% vs. 33%).

To date, a study on cross-presentation potential of chimeric T7 phage nanoparticles displaying a minimal CTL epitope has not been reported. Furthermore, a limited number of studies have investigated the effect of flanking residues on cross-presentation efficiency of and CTL induction by VNPs displaying a CTL polytope. To address this issue in our study, the T7-p66x2 phage nanoparticles were constructed and evaluated beside T7-p66 nanoparticles, to assess the role of alanine-alanine flanking sequences in cross-presentation of a p66 di-epitope (p66x2) when displayed on T7 phage capsids. The alanine-alanine spacer has shown a positive effect on correct processing and presentation of CTL polytopes encoded by a plasmid DNA (pDNA) [35], [36]. Although, alanine-alanine spacer has not been investigated in the context of a particulate carrier displaying a heterologous CTL polytope; however, other amino acid linkers have been explored in previous studies using VLP platforms. Yeast-derived Ty-VLPs carrying two different CTL epitopes linked by a glycine-glycine (GG) or glycine-serine (GS) spacer successfully elicited T cell responses against both epitopes [37]. The authors concluded that designing a flexible linker (GG or GS) helps correct cross-presentation of polytopes to immune system. In accordance, Rueda *et al*. showed that only parvovirus VLPs carrying two copies of the ovalbumin CTL epitope linked by two glycines were able to be properly processed [38]. However, in both studies a linker was only introduced between two CTL epitopes and both C- and N-terminal flanking residues were defined by VLP capsomer amino acid context. In contrast, in the T7-p66x2 phage nanoparticles and p66x2 synthetic peptide, two copies of the p66 CTL epitope were both linked and flanked C-terminally and N-terminally by a di-alanine spacer to create a similar context for both epitopes. Interestingly, only the T7-p66 chimer in which the p66 peptide was directly linked to 10B capsomer via a GGGS flexible spacer was successfully processed and cross-presented and induced effective anti-tumor CTL responses while the T7-p66x2 phage or the synthetic p66x2 peptide in which p66 peptide was linked to GGGS linker via a di-alanine spacer failed to be properly presented. These data supports the previous reports that glycine and serine flanking residues enhance cross-presentation of VLP-delivered CTL epitopes; even though whether cross-presentation of the polytopes delivered by VNPs can be universally improved with (glycine)_n_-serine spacers remains an open question. Furthermore, these data implicate that different flanking residues would behave differently when expressed endogenously by a polytope-encoding pDNA than when delivered by a particulate platform and enter cross-presentation pathway.

It has been hypothesized that pre-existing antibodies against carrier proteins can negatively affect the humoral immune response to the conjugated antigen [23], [24]. However, this phenomenon known as carrier-induced epitopic suppression (CIES) only has been a concern with antibody induction against B cell epitopes. In this study, to investigate whether cross-presentation of the p66 CTL epitope is suppressed by CIES phenomenon, we measured anti-T7 capsid IgG antibodies in mice sera. Despite a high titer of anti-T7 capsid IgG, no interference with induction of T cell responses against p66 epitope was observed. Furthermore, characterization of IgG isotypes showed a predominant induction of IgG_2a_, a Th_1_-biased subclass, over IgG_1_, a Th_2_-biased subclass. IFN-γ as a prototypical Th_1_ cytokine and IL-4 as a prototypical Th_2_ cytokine induces IgG_2a_ andIgG_1_ class-switching in B cells [39]. Vaccination of mice with chimeric T7 phage nanoparticles induced both T7 capsid-specific IL-4 and p66 peptide-specific IFN-γ as assessed by ELISA and ELISPOT respectively. Generation of both IgG_1_ and IgG_2a_ isotypes against T7 capsid further confirmed that T7 phage platform elicited both Th_1_ and Th_2_ cell responses. However, the predominance of IgG_2a_ subclass as well as induction of efficient p66-specific IFN-γ and CTL response indicated that T7 phage nanoparticles elicit a Th_1_-dominant response in BALB/c mouse strain which is well known to preferentially develop Th_2_ cell responses [40], [41]. Our results are in accordance with the data obtained by Thomas *et al*. using a λ phage-based gene and peptide delivery system [25]. They demonstrated that the general immune response to λ phage inoculation was dominated by a Th_1_ response as determined by IFN-γ and IL-4 concentrations and a higher level of IgG_2a_ antibodies. It would be suggested that recognition of pathogen-associated molecular patterns (PAMPs) in T7 phage structure such as non-methylated CpG motifs by TLR9 on DCs favors induction of Th_1_ responses. Undoubtedly, *ex vivo* analysis of APC stimulation such as DC maturation in presence of polymyxin B as an LPS inhibitor would be a valuable sensitive procedure to analyze and more characterize the immunostimulatory effects of our T7-based vaccine formulation. In conclusion, the high immunogenicity of peptides displayed in dense, repetitive arrays on T7 phage icosahedral capsids makes T7 phage a promising carrier for peptide/protein vaccines against infectious diseases and cancer. However, T7 phage has a limited tolerance for the size of the displayed peptides (up to 40 amino acids) when fused to 415 copies of 10B capsid protein. An alternative approach for delivery of larger polytopes and proteins would be the use of the mid-copy (up to 15) T7 phage vectors which allow display of large proteins (up to 1200 amino acids). Furthermore, formulation of T7 nanoparticles with a safe human adjuvant would compensate for the reduced protein copy number and reduce the dose needed for an effective immune response and improve the immunogenicity and protective potential.
